# Short communication: unique metabolic signature of proliferative retinopathy in the tear fluid of diabetic patients with comorbidities — preliminary data for PPPM validation

**DOI:** 10.1007/s13167-023-00318-4

**Published:** 2023-02-22

**Authors:** Martina Kropp, Eline De Clerck, Trong-Tin Kevin Steve Vo, Gabriele Thumann, Vincenzo Costigliola, Olga Golubnitschaja

**Affiliations:** 1 Experimental Ophthalmology, University of Geneva, 1205 Geneva, Switzerland; 2 Ophthalmology Department, University Hospitals of Geneva, 1205 Geneva, Switzerland; 3 European Medical Association, EMA, Brussels, Belgium; 4 Predictive, Preventive and Personalised (3P) Medicine, Department of Radiation Oncology, University Hospital Bonn, Rheinische Friedrich-Wilhelms-Universität Bonn, 53127 Bonn, Germany

**Keywords:** Predictive preventive personalised medicine (3P / PPPM), Health-to-disease transition, Diabetes mellitus, Complications and comorbidities, Proliferative diabetic retinopathy, Human blindness, Tear fluid, Metabolomics, Mass spectrometry, Molecular signature, Biomarker panel, Stress, Blood-retinal barrier, Mitochondrial impairment, Cell death, Metabolic and signalling shifts, Inflammation, Neovascularisation, Branch chain amino acids, Mitochondrial metabolism, Acylcarnitines, Amino acid related, Bile acids, Ceramides, Lysophosphatidyl-choline, Nucleobases, Phosphatidyl-cholines, Triglycerides, Cholesterol esters, Fatty acids

## Abstract

Type 2 diabetes (T2DM) defined as the adult-onset type that is primarily not insulin-dependent, comprises over 95% of all diabetes mellitus (DM) cases. According to global records, 537 million adults aged 20-79 years are affected by DM that means at least 1 out of 15 persons. This number is projected to grow by 51% by the year 2045. One of the most common complications of T2DM is diabetic retinopathy (DR) with an overall prevalence over 30%. The total number of the DR-related visual impairments is on the rise, due to the growing T2DM population. Proliferative diabetic retinopathy (PDR) is the progressing DR and leading cause of preventable blindness in working-age adults. Moreover, PDR with characteristic systemic attributes including mitochondrial impairment, increased cell death and chronic inflammation, is an independent predictor of the cascading DM-complications such as ischemic stroke. Therefore, early DR is a reliable predictor appearing upstream of this “domino effect”. Global screening, leading to timely identification of DM-related complications, is insufficiently implemented by currently applied reactive medicine. A personalised predictive approach and cost-effective targeted prevention shortly - predictive, preventive and personalised medicine (PPPM / 3PM) could make a good use of the accumulated knowledge, preventing blindness and other severe DM complications. In order to reach this goal, reliable stage- and disease-specific biomarker panels are needed characterised by an easy way of the sample collection, high sensitivity and specificity of analyses. In the current study, we tested the hypothesis that non-invasively collected tear fluid is a robust source for the analysis of ocular and systemic (DM-related complications) biomarker patterns suitable for differential diagnosis of stable DR versus PDR. Here, we report the first results of the comprehensive ongoing study, in which we correlate individualised patient profiles (healthy controls versus patients with stable D as well as patients with PDR with and without co-morbidities) with their metabolic profiles in the tear fluid. Comparative mass spectrometric analysis performed has identified following metabolic clusters which are differentially expressed in the groups of comparison: acylcarnitines, amino acid & related compounds, bile acids, ceramides, lysophosphatidyl-choline, nucleobases & related compounds, phosphatidyl-cholines, triglycerides, cholesterol esters, and fatty acids. Our preliminary data strongly support potential clinical utility of metabolic patterns in the tear fluid indicating a unique metabolic signature characteristic for the DR stages and PDR progression. This pilot study creates a platform for validating the tear fluid biomarker patterns to stratify T2DM-patients predisposed to the PDR. Moreover, since PDR is an independent predictor of severe T2DM-related complications such as ischemic stroke, our international project aims to create an analytical prototype for the “diagnostic tree” (yes/no) applicable to healthrisk assessment in diabetes care.

## Concerns about the status quo in type 2 diabetes (T2DM) management

According to WHO, within the period from 1980 to 2014, the number of diabetic patients increased 4-fold [https://www.who.int/news-room/fact-sheets/detail/diabetes]. From 2000 to 2019, the diabetes-related mortality increased by 3%, and in 2019, DM and DM-related kidney disease caused about 2 million deaths. T2DM, defined as adult-onset type of DM, comprises over 95% of all DM cases. Current reports indicate about 537 million adults (20–79 years old) affected by DM worldwide [1], which is equal to 1 out of 15 persons, and the number is expected to rise by 51% by the year 2045 [2, 3]. T2DM symptoms are slowly developing over months and years, whereas T1DM is characterised by a sudden onset of clear and severe lead symptoms. Consequently, the disease is frequently diagnosed only several years after onset, when DM complications are clinically manifest [4] such as diabetic retinopathy (DR), nephropathy, neuropathy, silent myocardial infarction, ischemic stroke, impaired wound healing, diabetic foot and cancers. To treat patients in this situation, with complications already present, is understood as reactive medicine (in contrast to preventive medicine). In average, annual medical expenditures of $16,752 per DM patient have been reported that translates in an annual global budget of $8.5 × 10^12^. In case that DM care and overall disease management remains “reactive”, by the year 2024, this annual budget will rise to $12 × 10^12^. Even economically strong countries will not be able to cover these costs. In addition, important social aspects will impose. Amongst the very diverse handicaps arising from DM complications, the physical and mental disability has a large impact on the quality of life (including family life as well as professional activity).

A paradigm shift from reactive medical services to cost-effective and patient-friendly PPPM approaches as proposed by the European Association for Predictive, Preventive and Personalised Medicine (EPMA) in the year 2009 [5–12] is pivotal to reverse current trends in DM care. Numerous research efforts have been made to promote PPPM strategies for DM risk prediction and personalised prevention at the level of pre-diabetes [13, 14]. Still, evidence-based predictive and preventive approaches are strongly underrepresented in daily medical practice in primary, secondary and tertiary DM care and specifically for prevention of DM complications in case of a positive diagnosis.

Around 30% of T2DM patients will develop DR, and is therefore the most frequent complication in T2DM [15]. DR is the leading cause of preventable blindness in adults aged 20–74 years, and although the portion of blinded DM patients is declining, the number of DR-related visual impairments is rising, due to increasing numbers of T2DM patients worldwide [16]. In the process, the proliferative form of diabetic retinopathy (PDR) is responsible for severe vision impairment and blindness [10]. Further, PDR demonstrates characteristic systemic attributes including mitochondrial impairment, increased cell death and chronic inflammation. This is an independent predictor for the cascade of subsequent DM complications such as ischemic stroke [17]. Unfortunately, early DR as the reliable predictor appearing upstream of the “domino effect” is neglected by the current practice of reactive medicine. It is therefore recommended to switch to a personalised predictive approach leading to a cost-effective targeted prevention called PPPM / 3P medicine [8].

## Evidence-based working hypothesis in the framework of 3P medicine

As previously published, tear fluid provides not only a unique source of information relevant for the prediction and prognosis of local but also systemic effects and chronic disorders [18]. Moreover, compared to other types of body fluid, tear fluid biomarker panels are known as particularly stable showing only neglectable influence by neither intraindividual lifestyle modifications (e.g. nutrition) or the time of the day. For this reason, tear fluid is highly relevant for predicting long-term effects such as a health-to-disease transition and disease progression in DM-associated complications [19]. Further, shifted metabolic pathways and bioenergetics, compromised mitochondrial health, microvascular deficits and small vessel disease, chronic inflammation and excessive tissue remodelling, all relevant for PDR pathogenesis and DM-related complications, are per-evidence reflected in human tear fluid metabolite patters [17, 18].

Here, we hypothesised that tear fluid biomarker panels are instrumental for the predictive stratification of DM patients with versus without comorbidities based on PDR-specific metabolic patterns. The study was designed to apply metabolomics by MS analysis to determine metabolic panels, which might be characteristic for PDR specifically in DM patients with comorbidities. The analysis of first study data presented here was focused on qualitative evaluation of the metabolites (present versus absent) in three different groups of patients versus healthy controls but not in a statistically significant quantification yet, that justifies well small numbers of patients and controls of the presented pilot study with the overall goal to create a tool for a “diagnostic tree” (yes/no) that will allow personalised treatment algorithms.

## Study design

The pilot research project (Proof-of-Principle study) aimed to collect tear fluid samples of diabetic patients and age- and gender-matched controls. By telephonic recruitment performed at the University of Geneva, diabetic patients were asked for the sample collection after their annual control visit, where eligibility criteria were verified and tear samples collected. Age- and gender-matched control participants were recruited at the University of Bonn. Samples were collected between April and July 2022, immediately stored in 1.5-mL Eppendorf tubes at − 80 °C. After coding, samples were sent blinded to Biocrates Life Sciences AG (Innsbruck, Austria, https://biocrates.com) for the comprehensive MS analysis of metabolic profiles.

### Inclusion and exclusion criteria

**Controls**. Twelve healthy individuals were recruited for the study with a parity of gender. The age ranged between 22 and 72 years (*n* = 6 males, *n* = 6 females) with at least one representative in each life decade from the third to the eight. All controls underwent a routine health check-up excluding major pathologies detected as an inclusion or exclusion criterion applied. Ophthalmological examinations were thoroughly performed. Any history of diabetes or eye disorder was an exclusion criterion. One sample from the right eye was collected per participant.

**Patients**. Six diabetic patients (5 males and 1 female) with DR (12 eyes) were recruited for the study sub-grouped as stable DR (*n* = 2), with PDR but no comorbidities (*n* = 1) and with both - PDR and comorbidities (*n* = 3). All patients were aged between 18 and 45 years and diagnosed with T2DM. Comorbidities were collateral pathologies such as chronic inflammation, impaired healing or cardiovascular diseases. No ophthalmic examinations were performed within the course of the study since all patients were under regular ophthalmologic control.

Additional exclusion criteria common for both, controls and patients, were pregnancy, acute infectious diseases, genetic diseases with impacts on glucose and insulin metabolism, genetic and non-genetic diseases and syndromes leading to systemic primary vascular dysregulation such as Flammer syndrome, and secondary vasospasm such as cancer patients under chemotherapy, neurodegenerative disorders like Alzheimer’s or Parkinson’s disease, the inability to refuse or give informed consent, a COVID-19 infection of the participant and/or close relatives, partners and friends within the last 4 weeks before the sample collection and a vaccination of the participant within the last 6 weeks before the sample collection.

### Tear fluid collection and preservation

Commercially available Schirmer tear strips (sterile diagnostic strips, individually packed, Aivimed GmbH, Wiesbaden, Germany) were used to collect tear fluid samples according to the protocols provided by the manufacturer. Briefly, the strips were inserted into both eyes over the lower lid margin, midway between the middle and outer third without anaesthesia. To avoid any sample contamination, the healthcare giver carried gloves and avoided to touch the cheek of the participants with the strips. The participant was then asked to close the eyes. After 1−5 min (depending on the collected volume), the wetting of the Schirmer paper was measured in mm and recorded individually. Immediately after the tear fluid was taken, each individual strip was placed into a sterile Eppendorf tube and stored at − 80 °C until MS analysis. 

### Mass spectrometry analysis

MS analysis was performed by Biocrates Life Sciences AG utilising the MxP®Quant 500 kit for metabolomics. For measuring metabolites’ concentration in the tear fluid, it was extracted from whole strips with 200 μL MeOH. The commercially available kit settled by Biocrates Life Sciences was used for the differential analysis of metabolites.

Lipids were measured by flow injection analysis-tandem MS (FIA-MS/MS) using a 5500 QTRAP® instrument (AB Sciex, Darmstadt, Germany) with an electrospray ionisation (ESI) source, and small molecules were measured by liquid chromatography-tandem MS (LC-MS/MS) using a 5500 QTRAP® instrument (AB Sciex, Darmstadt, Germany). The experimental metabolomic measurement technique is described in detail by patents EP1897014B1 and EP1875401B1 (https://patents.google.com/patent/EP1897014B1 and https://patents.google.com/patent/ EP1875401B1). Briefly, a 96-well based sample preparation device was used to quantitatively analyse metabolite profiles in all samples. The device consists of inserts, which have been impregnated with internal standards, and a predefined sample amount was added to the inserts. Next, a phenyl isothiocyanate (PITC) solution was added to derivatise some of the analytes (e.g. amino acids). Next, the target analytes were extracted with an organic solvent followed by a dilution step. The obtained extracts were then analysed by FIA-MS/MS and LC-MS/MS using multiple reaction monitoring (MRM) to detect the analytes. Individual metabolite concentrations were calculated using the Siex Analyst® and the Waters MassLynx® softwares; the data were imported into the Biocates’ MetlDQ™ software for further analysis. The accuracy of the measurements, determined with the accuracy of the calibration, was in the normal range of the method for all analytes. Quality control samples were within the predefined tolerances of the method.

## Results and data interpretation

According to the working hypothesis (see above), the study was designed for detecting metabolic patterns which are characteristic for the PDR. The study was focused on the metabolites’ qualification (present versus absent) in stratified patients, in order to create the tool prototype for the diagnostic tree (yes/no).

### Metabolic panels specific for the DR

**The following metabolites were absent in healthy controls but detected in DR patients:**

Metabolite cluster *acylcarnitines*: butenylcarnitine, glutaconylcarnitine, pimelylcarnitine and octadecadienoylcarnitine

Metabolite cluster *amino acids & related compounds*: betaine, 5-aminovaleric acid

Metabolite cluster *bile acids*: glycolithocholic acid sulfate

Metabolite cluster *ceramides*: ceramide (d18:1/18:0(OH))

Metabolite cluster *lysophosphatidyl-cholines*: lysophosphatidyl-choline a C24:0, C26:0, C26:1 and C28:0

Metabolite cluster *nucleobases related compounds*: xanthine

Metabolite cluster *phosphatidyl-cholines*: phosphatidyl-choline aa C24:0, C26:0, C32:3, C34:3, C34:4, C42:1, C42:2 and C42:5, phosphatidyl-choline ae C30:0, C38:0, C40:4, C40:6 and C44:5

Metabolite cluster *triglycerides*: triacylglyceride (16:1_36:1).

**The following metabolites were detected in healthy controls but were absent in all DR patients:**

Metabolite cluster *acylcarnitines*: decadienoyl-carnitine

Metabolite cluster *bile acids*: glycoursodeoxy-cholic acid, taurochenodeoxy-cholic acid and taurodeoxycholic acid

Metabolite cluster *lysophosphatidyl-cholines*: lysophosphatidyl-choline a C14:0 and C20:3

Metabolite cluster *triglycerides*: triacylglyceride (18:1_36:1), triacylglyceride (18:2_36:0), triacylglyceride (18:2_38:6), triacylglyceride (20:5_36:2) and triacylglyceride (22:5_34:2).

### Unique metabolic signature of proliferative retinopathy

Preliminary data of a unique metabolic signature for DR patients with first hints for a specific profile for PDR patients is summarised in Table 1.

**Table 1:** Proposed metabolic signature of PDR in the tear fluid of DR patients: comparative analysis of individual metabolites in DR patients stratified by non-proliferative DR versus PDR and DM-related comorbidities (with versus without); qualitative (detected “yes” versus undetected “no”) characterisation was performed using the detection limit (DL) of corresponding metabolites

Metabolite	DL (μM)	Healthy individuals: metabolite detected (no/yes against DL)	DM patients with DR: metabolite detected (no/yes against DL)	DM patients with PDR without comorbidities: metabolite detected (no/yes against DL)	DM patients with PDR and comorbidities: metabolite detected (no/yes against DL)
Metabolite cluster: acylcarnitines					
Dodecanoyl-carnitine	0.018	Yes (> DL)	Yes (> DL)	No	No
Tetradecanoyl-carnitine	0.017	Yes (> DL)	Yes (> DL)	No	No
Tetradecenoyl-carnitine	0.008	Yes (> DL)	Yes (> DL)	No	No
Metabolite clusters: amino acids & related compouns					
Asymmetric dimethylarginine	0.080	Yes (> DL)	Yes (> DL)	No	No
Methionine-sulfoxide	0.10	Yes (> DL)	Yes (> DL)	No	No
Metabolite cluster: cholesterol esters					
Cholesteryl ester 15:1	0.15	Yes (> DL)	Yes (> DL)	No	No
Metabolite cluster: fatty acids					
Eicosadienoic acid	0.80	Yes (> DL)	Yes (> DL)	No	No
Metabolite cluster: triglycerides					
Triacylglyceride (14:0_34:0)	0.079	Yes (> DL)	Yes (> DL)	No	No
Triacylglyceride (16:0_32:0)	0.26	Yes (> DL)	Yes (> DL)	No	No

Accumulated abundant evidence indicates that small non-protein metabolites are stably altered in individuals with T2DM. Further, their profiles might be characteristic for pre-diabetic stages and progression of T2DM and associated pathologies [18]. Consequently, the body fluid (such as blood plasma and tear fluid) metabolomics is considered of potential clinical utility for the patient stratification, disease prediction, targeted prevention and personalisation of treatment algorithms in primary, secondary and tertiary DM care.

Herewith, we correlate our findings in the tear fluid with an accumulated knowledge about metabolite clusters, yet qualified and quantified generally in blood.

### Metabolite cluster: acylcarnitines

Carnitine metabolite patterns are known as being altered in blood of DR patients. Moreover, these metabolic profiles demonstrate qualitative and quantitative specificity for T2DM patients with DR, versus without retinopathy as well as patients with PDR versus non-proliferative DR [20]. The carnitine shuttle is an essential attribute of the mitochondrial metabolism. Contextually altered carnitines profile is an independent indicator of mitochondrial stress and compromised mitochondrial health. Carnitine profile measurements are instrumental for identifying the mitochondrial dysfunction associated pathologies such as diabetes, CVD and cancers, amongst others [20]. Due to disease-specific carnitine metabolite set-ups, elevated plasma levels of some acylcarnitine metabolites were associated with increased cardiovascular risks in T2DM patients.

In our analytical sets, stage/disease-specific acylcarnitine metabolites were distinguishable in the tear fluid of the DR against healthy controls as well as in the PDR associated with DM-related complications.

### Metabolite clusters: amino acids and related compounds

Plasma and serum amino acid profiles considered reliable biomarker panels for population screening and predictive diagnostics of DM and related complications, since glucose and amino acid metabolisms are closely connected [21]. Meta-analytical studies associate an increased T2DM risks with upregulated blood plasma and serum concentrations of branched-chain, aromatic, alanine, glutamate, lysine and methionine which were co-detected with enhanced concentrations of carbohydrates, energy-related metabolites as well as some acylcarnitines, triacylglycerols, (lyso) phosphatidylethanolamines and ceramides [22]. These are promising patterns for the T2DM prediction. Further, innovative concepts of a dietary amino acid (AA) composition have been proposed to improve the overall quality of dietary AA compositions in targeted T2DM prevention [23].

Our analyses let assume 5-aminovaleric acid and betaine distinguishable specifically in the tear fluid of the DR patients.

### Metabolite cluster: lysophosphatidyl-cholines

Lysophosphatidylcholines (LPC) are potent inflammatory lipids: significantly increased LPC blood contents correlate well with neurodegeneration and DR progression from the pre-proliferative stage into severe disease [24]. The underlying PDR pathomechanisms involve LPC into blood-retinal barrier damage by promoting oxidative stress injury via TLR4/NF-*κ*B signalling. Further, LPC was proposed to promote pro-angiogenic reactions via VEGFR2 impacting retinal vascular endothelium and inducing vasopermeability [25]. Inhibition of LPC might prevent DM-mediated blood-retinal barrier dysfunction and uncontrolled angiogenesis-related diseases [26].

In our analytical sets, lysophosphatidylcholine profiles detected in the tear fluid were specific for the DR and differed substantially from metabolic profiles of healthy controls.

### Metabolite clusters: nucleobases and related compounds

In our analytical sets, xanthine has been found specifically in DR patients while being absent (below the detection limit) in healthy controls. Xanthine is a well-known intermediate product of the purine nucleotide catabolism with urate as the end product and thus found in multiple mammalian body fluids [27]. Though it can bind in vitro to all four nucleotide bases, naturally, it does not incorporate to DNA or RNA due to its instability amongst other reasons [27]. However, oxidative deamination of DNA can lead to deoxyxanthosine from deoxyguanosine produced by reactive nitrogen species as a consequence of oxidative and nitrosative stress [27]. Nguyen et al. reported 40-fold increased levels of xanthine residues in DNA of human lymphoblastoid TK6 cells after exposure to NO [28]. There is evidence that insulin resistance in T2DM might induce the synthesis and deposition of advanced glycation end products, reactive oxygen species and reactive nitrogen species, leading to cellular stress, protein and mitochondrial dysfunction and metabolic syndrome, inflammation and apoptosis [29].

### Metabolite clusters: fatty acid and triglycerides

Lipids and lipoproteins significantly contribute to the pathogenesis of DR. Intervention studies with omega-3 fatty acids demonstrate protective effects against the development and progression of DR. However, the role and impact of individual lipid classes remain underexplored [30]. To this end, association between the hypertriglyceridemia in blood of DM patients and the lower-extremity amputation risk has been demonstrated [31], which is a complication downstream towards the DR in the overall cascade of the DM-related complications [17]. Further, blood triglycerides have been demonstrated as a more reliable pre-diabetes biomarker and predictor compared to the total cholesterol, LDL, HDL, LDL/HDL ratio, cholesterol/HDL ratio and LDL/HDL ratio [32].

In our analytical sets, triglyceride profiles detected in the tear fluid were evidently specific for the PDR and differed substantially between healthy controls and DR patients. Further, the absence of eicosadienoic acid was specific for the PDR.

### Metabolite cluster: bile acids

Bile acids play an important role in regulating glucose, energy and lipid-associated metabolic pathways. Contextually, metabolic diseases such as obesity, dyslipidemia and T2DM have been associated with dysregulated bile acid homeostasis [33]. Moreover, bile acids are involved in a control over inflammation regulation via signalling pathway based on the nuclear farnesoid X receptor and Takeda G protein-coupled receptor 5. Bile acid signalling and the gut microbiome have been proposed as the targets for treating diabetes. To this end, bile acids have been proposed as a protective factor against DR with great therapeutic potential in the retinal disorder management [34–36].

In our analytical sets, specifically the glyolithocholic acid sulfate belonging to the metabolite cluster of bile acids was detected in DR patients but absent in healthy controls, while glycoursodeoxy-cholic acid, taurochenodeoxy-cholic acid and taurodeoxycholic acid were detected in healthy controls but absent in DR patients.

### Metabolite cluster: ceramides

Via their lipotoxicity, ceramides contribute to the diabetogenic effects implicated in inflammation, β cell apoptosis and insulin resistance, and are involved in the initiation and progression of microvascular and macrovascular complications of DM relevant for cardiovascular disease, blindness, nephropathy, peripheral neuropathy and lower-limb amputation [37–39].

In cultured cells and isolated tissues, ceramides promote mitochondrial dysfunction, disrupted vasodilatation and apoptosis [40]. Accumulated research data indicate that the targeted reduction of the ceramide biosynthesis and their decreased circulating levels is beneficial for preventing diabetes and DM-related complications [38]. To this end, serum ceramides are considered reliable biomarkers of adverse cardiovascular disease outcomes. Pre-clinical studies demonstrated protective affects against DM and CVD in mice and rats treated with ceramides inhibitors.

In our analytical sets, some ceramide fractions were evidently more specific for the DR patients.

### Metabolite cluster: phosphatidyl-cholines

Phosophatidyl-cholines are essential in controlling levels of circulating lipoproteins like very low-density (VLDL) and high-density (HDL) lipoproteins [40]. They are phospholipid components of the class of plasma lipoproteins and needed for lipoprotein assembly and secretion. Multiple studies demonstrated a positive association of increased levels of phosphatidyl-cholines with obesity, metabolic syndrome, insulin resistance and gestational diabetes [40, 41].

In the eye, choline and phosphatidyl-choline are factors included in tears and the meibum. In the retina, a choline deficiency is associated with a distorted differentiation of retinal neuronal cells and cytoarchitectural defects. Moreover, increased phosphatidyl-choline levels are linked to lowered risks of diabetes and cardiovascular diseases [42].

Interestingly, our analytical sets demonstrated the presence of some specific phosphatidyl-choline fractions in DR patients, which were absent in healthy controls.

### Metabolite cluster: cholesterol esters

Ample data are available regarding the cholesterol implication in the pathophysiology of cardiovascular diseases, and cholesterol ester-enriched foam cells are an indicator of atherosclerotic plaques [43]. It is also well established that T1DM and T2DM patients have an increased probability of developing cardiovascular disease as a severe diabetes-related complication caused by augmented cholesterol levels [44]. However, even after lowering cholesterol levels, a residual risk remains, which has been associated to remnant lipoprotein particles (RLP) [44]. Chait et al. defined RLPs as postlipolytic partially triglyceride-depleted particles, and the longer RLPs remain in circulation, the greater their enrichment with cholesteryl esters [44]. Cholesterol esters are involved in multiple metabolic processes in the body [45]. In the retina, mainly nonesterified cholesterol is found in a well-controlled homeostasis, and reduced cholesterol levels lead to retinal degeneration [45]. Its role in DR is still not fully understood, but multiple studies support a positive correlation between dysregulated lipid metabolism and DR [45].

Our analytical sets demonstrated an absence of cholesterol esters in the PDR (with and without comorbidities), while being present in DR patients and healthy controls.

## Conclusions and outlook in the framework of 3P medicine

As a diagnostic tool, the tear fluid advantages over other bodily fluids due to non-invasive approach, reflection of systemic affects as well as stability and specificity of biomarker panels [18]. Tear fluid is rich in metabolites associated with signalling pathways, abnormal stress reactions, metabolic and bio-energetic shifts, compromised mitochondrial health, microvascular deficits, systemic inflammation and apoptosis, amongst others—all involved in pre-diabetes and cascading diabetes-related complications [17, 18]. Contextually, tear fluid metabolomics is of great clinical utility for stratifying patients, predicting the health-to-disease transition and targeting cost-effective prevention and individualised treatments in advanced primary and secondary pre-diabetes care.

In the current study, we verified the hypothesis that the tear fluid biomarker panels are instrumental for stratifying PDR patients as a particularly severe complication of T2DM. Since specifically PDR is an independent predictor of severe T2DM complications such as ischemic stroke [46], the main concept of our international project is to create the tool prototype for the diagnostic tree (yes/no) crucial for the robust predictive and preventive approach.

In our analytical sets, the following metabolite clusters are per-evidence involved in the unique metabolic signature of DR and PDR in the tear fluid of diabetic patients: *acylcarnitines*, *amino acids and related compounds*, *bile acids*, *ceramides*, *lysophosphatidyl-choline*, *nucleobases and related* *compounds*, *phosphatidyl-cholines*, *triglycerides*, *cholesterol esters* and *fatty acids*. Corresponding molecular panels detailed above are instrumental for the patient stratification towards the disease severity and individualised treatment algorithms to be implemented. Presented achievements should get validated in extended analytical sets. Cost-effective primary and secondary prevention is envisaged based on the validated diagnostic targets for both DR and PDR with comorbidities. Depending on the individually affected metabolic pathways and sub-cellular structures, corresponding mitigation and preventive measures will be focused on

- Microvascular deficits and small vessels disease [17, 47, 48]
- Adequate stress reactions [12, 46, 49, 50]
- Systemic inflammation and impaired wound healing [51, 52]
- Mitochondrial health sustainability and bioenergetics [46, 50, 53–55], amongst others.

## Study limitations

In the presented pilot study, comparative analyses considered metabolic shifts in stratified diabetic patients with clinically manifested DR but not in DM patients without DR—the target group which should be included into the follow-up validation project. Further, current study was focused on the qualification (yes/no) of metabolites in stratified groups but not on the quantification of metabolites which should be performed in the follow-up multicentre validation study planned.

## Abbreviations

DM: Diabetes mellitus
DR: Diabetic retinopathy
PDR: Proliferative diabetic retinopathy
PPPM/3PM: Predictive, preventive and personalised medicine
T1DM: Diabetes mellitus type 1
T2DM: Diabetes mellitus type 2
MS: Mass spectrometry
RLPs: Remnant lipoprotein particles
